# RNA-Seq analysis of gene expression in 25 cases of canine lymphoma undergoing CHOP chemotherapy

**DOI:** 10.1186/s13104-022-06003-5

**Published:** 2022-03-22

**Authors:** Miles W. Mee, Sydney Faulkner, Geoffrey A. Wood, J. Paul Woods, Dorothee Bienzle, Brenda L. Coomber

**Affiliations:** 1 Department of Biomedical Sciences, Ontario Veterinary College, University of Guelph, Guelph, ON N1G 2W1 Canada; 2 Department of Pathobiology, Ontario Veterinary College, University of Guelph, Guelph, ON N1G 2W1 Canada; 3 Department of Clinical Studies and Mona Campbell Centre for Animal Cancer, Ontario Veterinary College, University of Guelph, Guelph, ON N1G 2W1 Canada

**Keywords:** Cancer, Lymphoma, Dog, Gene expression, Comparative oncology, Chemotherapy, RNA sequencing

## Abstract

**Objectives:**

Canine lymphoma, the most common hematological cancer in dogs, shares many molecular and clinical characteristics with human Non-Hodgkin lymphoma (NHL). The standard treatment for canine lymphoma is “CHOP” multiagent chemotherapy protocol consisting of Cyclophosphamide, Doxorubicin (Hydroxydaunorubicin), Vincristine (Oncovin™), and Prednisone. Approximately 70–85% of patients treated with CHOP achieve clinical remission. However, duration of remission varies and the majority of dogs eventually relapse. To identify possible biomarkers for patients failing to achieve remission, we performed RNA-Seq analysis on 25 cases of canine lymphoma obtained prior the start of their CHOP therapy regime and assessed gene expression associated with patient progression free survival (PFS).

**Data description:**

The data consists of (1) raw RNA-Seq reads in 75 bp fastq format from fine needle aspirate samples of enlarged lymph nodes from canine patients with naturally occurring lymphoma; (2) Fragments Per Kilobase Million (FPKM) values for each sample; (3) raw transcript counts for each sample; (4) anonymized patient details including PFS; (5) heat map of gene expression and (6) Cox proportional hazard analysis showing significantly expressed genes. These data may be useful for comparative analysis of gene expression in human NHL and analysis of gene expression associated with disease outcome in canine lymphoma.

## Objective

Canine lymphoma is the most common haematopoietic neoplasm in dogs [1] and is similar to human non-Hodgkin lymphoma (NHL). Both canine and human NHL have similar clinical presentation, molecular biology, therapy, and treatment response [2, 3]. Lymphoma is also common in dogs, and is treated with similar multiagent chemotherapy regimens as human disease yet has a much faster disease progression (time to relapse of 6–8 months is typical [4]) than that seen in NHL. This makes canine lymphoma an attractive comparative oncology model for the most aggressive human NHL cases.

The standard treatment is standard in both canine lymphoma and NHL, consisting of a multi-agent chemotherapy protocol of cyclophosphamide, doxorubicin, vincristine, and prednisone (CHOP), often with the addition of rituximab in humans [5]. There is a high initial response rate for canine lymphoma to CHOP of about 70% to 85%. However, the duration of remission varies and the majority of patients will eventually relapse [4, 6]. The failure of many canine lymphoma patients to achieve long term remission has raised interest in developing methods to predict their response to CHOP therapy. While several molecular biomarkers have been proposed [7–9] in general they lack a clear therapeutic target, thus additional research is required. The data provided here may be useful for analysis of gene expression changes related to disease outcome in canine lymphoma, and for comparative analysis of gene expression in human NHL.

## Data description

### Case enrolment

Dogs with naturally occurring lymphoma, diagnosed by cytology or histology at the Mona Campbell Centre for Animal Cancer, University of Guelph, who had received no prior treatment other than a single injection of prednisone were eligible for this study. No breed, sex or age restrictions were in place, but dogs with other concurrent neoplasms or prior neoplasms including lymphoma were excluded. Lymphomas were immunophenotyped by flow cytometry [10]. Dogs were enrolled with the intention to treat with standard CHOP therapy and were monitored by physical examinations ± diagnostic imaging for a minimum of 6 months to categorize response as complete remission or not by WHO criteria [11]. Dogs lost to follow-up, or not progressed at last check were censored for PFS.

### RNA-Seq data generation and processing

Tumor samples were collected by fine needle aspirate and expressed into sterile collection vials containing 1.0 ml of RNAprotect Cell Reagent (Qiagen). Poly A-RNA was isolated from the tissue samples using QIAGEN RNeasy isolation kit and quantified using a Nanodrop spectrophotometer. The RNA integrity number (RIN) was calculated for each sample using Bioanalyzer analysis. Twenty-five samples with RIN above 9 were selected for sequencing, including 16 immunophenotyped as B-cell lymphoma, three immunophenotyped as T-cell lymphoma, and six patients with missing immunophenotype. The selected samples were sequenced using the Illumina NextSeq platform by the London Regional Genomics Centre, London, ON, Canada. The sequence data were returned in single read 75 bp fastq format. Data Set 1 contains the RNA sequencing results for this publication, which have been deposited in NCBI’s Gene Expression Omnibus and are accessible through GEO Series accession number GSE130874 [12].

The RNA-Seq reads were assessed for quality using ***FastQC*** [13]. The reference genome (CanFam3.1) and gene model (CanFam3.1.88.gtf) were downloaded from Ensembl [14]. The raw fastq reads were aligned to the CanFam3.1 reference sequence and CanFam3.1.88.gtf annotation file using ***Hisat2*** [15]. ***StringTie*** [16] was used to annotate genes and quantify their expression. Fragments Per Kilobase Million (FPKM) values were calculated using the R package ***Ballgown*** [17] and are available in Data file 1 [12]. Raw transcript counts for expressed genes are available in Data file 2 [12]. Key patient details, including PFS are available in Data file 3 [18]. When we hierarchically clustered and visualized the RNA-Seq data from all sequenced samples, we observed two patient clusters corresponding to samples isolated by separate technicians, suggesting some batch effects existed between the patient clusters (Data file 4 [18]). While there were 1052 genes with unadjusted Cox Proportional Hazard model *p*-values < 0.05 (Data file 5 [18]), we were unable to confirm their significance in a validation set of cases. Thus, no research publication from this study was feasible. However, we feel the available data may be of use to others who are researching lymphoma in dogs and in humans (see Table 1).

**Table 1:** Overview of data files/data sets

Label	Name of data file/data set	File types (file extension)	Data repository and identifier (DOI or accession number)
Data set 1	Raw sequence reads for 25 canine lymphoma cases	FASTQ files (.fastq.gz)	NCBI GEO; https://identifiers.org/geo:GSE130874 [12]
Data file 1	FPKM values for expressed genes	TSV file (.tsv.gz)	NCBI GEO; https://identifiers.org/geo:GSE130874 [12]
Data file 2	Raw transcript counts for expressed genes	TSV file (.tsv.gz)	NCBI GEO; https://identifiers.org/geo:GSE130874 [12]
Data file 3	Clinical outcome for 25 canine lymphoma cases	TAB file (.tab)	Harvard Dataverse; https://doi.org/10.7910/DVN/PGL3BO [18]
Data file 4	Heatmap of gene expression	Adobe Acrobat portable document format file (.pdf)	Harvard Dataverse; https://doi.org/10.7910/DVN/PGL3BO [18]
Data file 5	Results of Cox proportional hazard analysis of gene expression	TAB file (.tab)	Harvard Dataverse; https://doi.org/10.7910/DVN/PGL3BO [18]

## Limitations

This study is limited by the small sample size, and technical issues resulting in batch effects further reducing the statistical power. The lack of complete immunophenotype information for all cases, and the lack of technical replicates (repeat sequencing) are also limitations. The selection of cases to sequence was initially influenced by client decision to treat their dogs with CHOP, and client consent to participate in this study. Recovery of sufficient total RNA of suitable quality from available cases and finances limited the RNA-Seq analysis to 25 samples. All or any of the above may have had unintended and unknown influence on the data obtained.

## Data Availability

The raw RNA sequencing results for this publication are available from the NCBI Gene Expression Omnibus and are accessible through GEO Series accession number GSE130874 https://identifiers.org/geo:GSE130874. The FPKM values and raw transcript counts are also available through GEO accession number GSE130874. The data on patient outcome, information on batch effects, and the results of a Cox Proportional Hazard evaluation are available at https://doi.org/10.7910/DVN/PGL3BO. Please see Table 1 and references [12, 18] for details and links to the data.

## Abbreviations

CHOP: Cyclophosphamide, Doxorubicin (Hydroxydaunorubicin), Vincristine (Oncovin™), Prednisone
NHL: Non-Hodgkin Lymphoma
FPKM: Fragments per kilobase of exon per million fragments mapped
PFS: Progression free survival
RIN: RNA integrity number
NCBI: National Center for Biotechnology Information
GEO: Gene Expression Omnibus
